# Multimodal pathophysiological dataset of gradual cerebral ischemia in a cohort of juvenile pigs

**DOI:** 10.1038/s41597-020-00781-y

**Published:** 2021-01-07

**Authors:** Martin G. Frasch, Bernd Walter, Christophe L. Herry, Reinhard Bauer

**Affiliations:** 1 University of Washington School of Medicine, Center on Human Development and Disability, Seattle, WA USA; 2 Department of Spine Surgery and Neurotraumatology, SRH Waldklinikum, Gera, Germany; 3 Institute of Molecular Cell Biology, Jena University Hospital, Jena, Germany; 4 Dynamical Analysis Lab, Ottawa Hospital Research Institute, Ottawa, ON Canada

**Keywords:** Electrocardiography - EKG, Electroencephalography - EEG, Neurophysiology, Brain injuries

## Abstract

Ischemic brain injuries are frequent and difficult to detect reliably or early. We present the multi-modal data set containing cardiovascular (blood pressure, blood flow, electrocardiogram) and brain electrical activities to derive electroencephalogram (EEG) biomarkers of corticothalamic communication under normal, sedation, and hypoxic/ischemic conditions with ensuing recovery. We provide technical validation using EEGLAB. We also delineate the corresponding changes in the electrocardiogram (ECG)-derived heart rate variability (HRV) with the potential for future in-depth analyses of joint EEG-ECG dynamics. We review an open-source methodology to derive signatures of coupling between the ECoG and electrothalamogram (EThG) signals contained in the presented data set to better characterize the dynamics of thalamocortical communication during these clinically relevant states. The data set is presented in full band sampled at 2000 Hz, so the additional potential exists for insights from the full-band EEG and high-frequency oscillations under the bespoke experimental conditions. Future studies on the dataset may contribute to the development of new brain monitoring technologies, which will facilitate the prevention of neurological injuries.

## Abstract (web summary)

**Table:** 

Measurement(s)	EEG with persistent abnormal rhythmic activity • electrocardiogram • Electrothalamogram • cerebral blood flow measurement • positive regulation of heart rate involved in baroreceptor response to decreased systemic arterial blood pressure • cerebral metabolic rate of oxygen • cerebral perfusion pressure
Technology Type(s)	electrophysiology assay • extracellular electrophysiology recording assay • fluorescent colormicrospheres • Electrophysiology
Factor Type(s)	sedation • cerebral ischemia • recovery
Sample Characteristic - Organism	Sus scrofa
Sample Characteristic - Environment	laboratory environment

Machine-accessible metadata file describing the reported data: 10.6084/m9.figshare.13251176

## Background & Summary

Surface EEG contains information about corticothalamic communication, which can be quantified even without invasive insertion of thalamic electrodes^1,2^. In this study on juvenile pigs, we introduced the basic stereotaxic approach to chronically recording electrothalamogram (EThG) and quantifying the effects of isoflurane and fentanyl sedation on the brain electrical activity from a ten-channel electrocorticogram (ECoG), EThG, and the cerebral blood flow^2^, followed by the characterization of the effects of gradual propofol sedation on these parameters^1^.

The choice of this animal model is dictated by its amenability to complex stereotactic chronic instrumentation, monitoring studies of sedation, and clinically relevant patterns of hypoxic/ischemic injury in a relatively large and gyrencephalic brain^2^.

Here, we present the unique data set underlying the above experiments and expand it to include the conditions of gradual ischemia and recovery that were part of the original experiments but were never presented before. We focus on the spontaneous ECoG/EThG activity and exclude the evoked potential responses from this dataset. We believe this makes for a focused presentation of this complex multimodal recording series and will present the evoked potentials dataset separately. By way of technical validation, we present a basic approach to derive ECoG/EEG biomarkers of normal, sedation, and hypoxic/ischemic conditions using EEGLAB^3^; we present an elementary approach to quantify the ECoG activity on the cohort level identifying state-specific independent component (IC) features of ECoG common to all animals studied.

Moreover, for the first time, we present the electrocardiogram (ECG) dataset accompanying the ECoG/EThG providing its basic technical validation, annotating, and providing the corresponding cardiovascular and brain regional and systemic continuous blood flow data.

We hope the present data set will lay the foundation for new brain monitoring technologies, which will facilitate the prevention of neurological disorders.

All experiments were carried out in accordance with the European Communities Council Directive 86/609/EEC for animal care and use. The Animal Research Committee of the Thuringian State government approved laboratory animal protocols.

## Methods

### Instrumentation

#### General instrumentation

Using a protocol approved by the Committee of Animal Care and Use of the Thuringian State Government (Germany) 11 mixed breed female pigs (7-weeks-old, 14.9 ± 1.2 kg BW) were instrumented as published^2^. Animals were anesthetized with 70% nitrous oxide in 30% oxygen and 2 Vol-% isoflurane (Vapour 19.3, Draegerwerk AG, Lübeck, Germany). Muscle relaxation was achieved with pancuronium bromide (0.4 mg/kg b.w./h, i.v.). Ventilation was performed in a pressure-controlled mode and was controlled by end-expiratory CO_2_ monitoring (Servo Ventilator 900 C; Siemens-Elema, Solna, Sweden) and hourly arterial blood gas analysis (ABL50 Blood Gas Analyzer and Hemoxymeter OSM2, Radiometer, Copenhagen, Denmark). Body temperature was maintained at 37.5 ± 0.5 °C. The urinary bladder was punctured and drained. Arterial blood pressure (ABP) was monitored continuously using catheters that were introduced into the abdominal aorta via the saphenous arteries. Arterial and central venous catheters (inserted into jugular veins) were connected with pressure transducers (P23Db, Statham Instruments, Puerto Rico) and continuously recorded.

A left thoracotomy was then performed through the third intercostal space. The pericardium was carefully opened, and a cerclage of plastic-coated wire was performed around the trunk of the pulmonary artery to appropriately adjust the trunk diameter for cardiac output control during the induction and maintenance of gradual ischemia, as described previously^4^. A catheter was also inserted into the left atrium for colored microsphere injection^5,6^ in order to measure cardiac output and regional cerebral blood flow.

ECG was recorded as Lead I using stainless steel needle electrodes inserted into both forelegs and hindleg (HSE EKA-PULS, Hugo Sachs Elektronik, March-Hugstetten, Germany). Physiological parameters were recorded together with brain electrophysiological data and the intracranial pressure (ICP, see below) with a multi-channel recording device (GJB Datentechnik Bolten & Jannek GbR, Ilmenau, Germany).

#### Instrumentation of the head

**Fig. 1:**
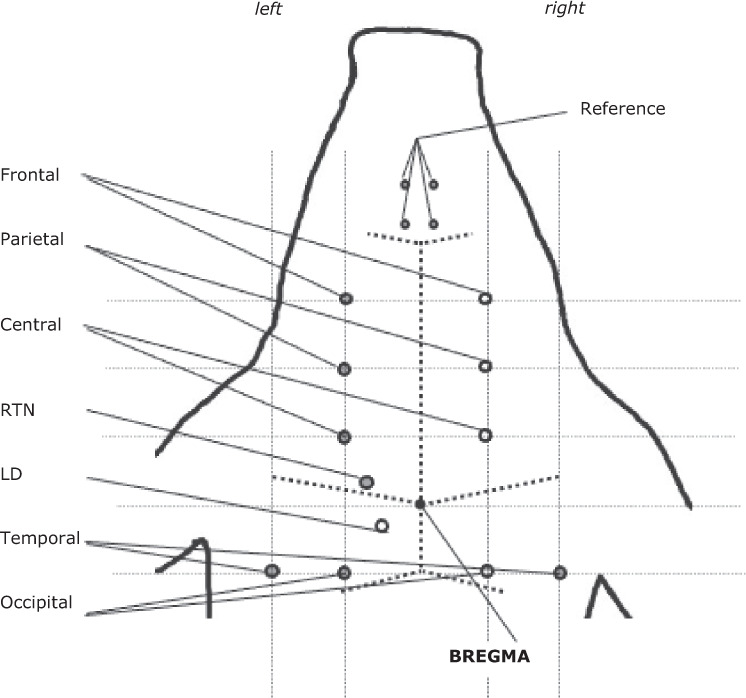
Instrumentation of pig for recording ECoG and EThG. For details see Table 1.

The animals were placed in a sphinx position with the head fixed in a stereotactic frame (Kopf Instruments, Tujunga, USA) (Fig. 1). The skull was exposed and burr holes were made for the insertion of ten electrocorticographic electrodes for bilateral leads from frontal, parietal, central, temporal, and occipital regions and guides for electrodes into the left thalamus, specifically, the reticular (RTN) and dorsolateral (LD) nuclei (unipolar recordings; reference, nasal bone).

The stereotactic coordinates of thalamic electrodes (RTN, AP + 2 mm, L 9 mm, V 24 mm, and LD, AP −2 mm, L 5 mm, V 20 mm, respectively) were predetermined in three pigs of the same age based on own experiences^2^ and an atlas reference^7^. Using a micromanipulator (Kopf Instruments, Tujunga, USA) guides for thalamic electrodes were inserted (Ø 0.9 mm), followed by insertion of the insulated unipolar silver/silver chloride electrodes. The length of the recording tip of the depth electrodes amounted to 2 mm. As reference served 4 connected electrodes inserted into the nasal bone. A ground electrode was placed in the back of the neck.

A further hole was drilled into the left parietal bone and a fiberoptic catheter was implanted into the subcortical white matter for intracranial pressure (ICP) measurements (Camino Laboratories, San Diego, USA).

A catheter was inserted into the superior sagittal sinus through a midline burr hole (Ø 3 mm, AP −4 mm), and advanced to the confluence of the sinuses to obtain brain venous blood samples.

The burr holes were sealed with bone wax and covered with dental acrylic resin in order to fix electrodes, probes, and catheters in place throughout the experiment.

RTN was chosen as a non-specific nucleus with a high density of GABA A receptors responsible for the sedative effects of propofol^8,9^ and gating influence on thalamocortical oscillations^10,11^, while LD represented a specific exteroceptive nucleus. Between RTN, LD, and eight ECoG channels we captured representative specific and non-specific thalamocortical communication.

### Description of experimental stages

**Fig. 2:**
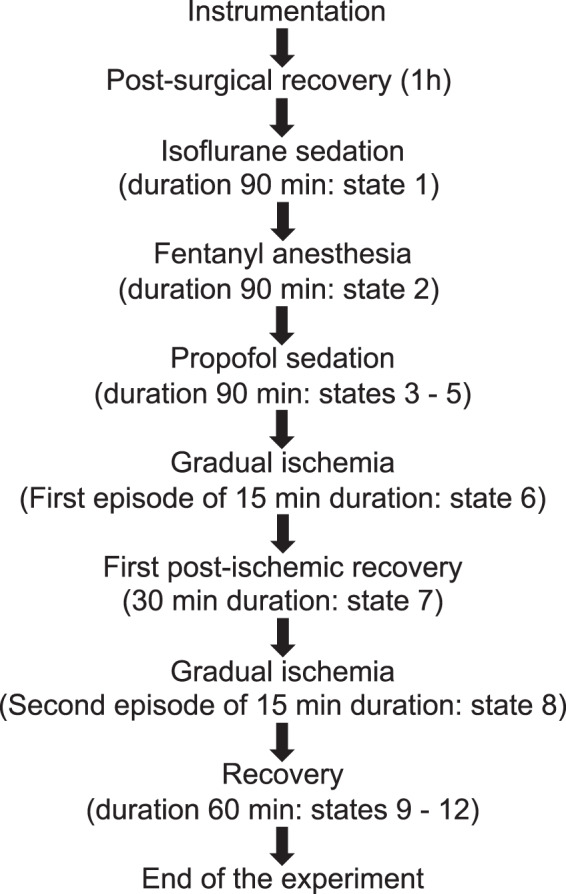
Experimental protocol of pig model of sedation and gradual ischemia.

The experimental protocol is summarized in Fig. 2. After post-surgical recovery, the pigs were allowed 90 minutes to stabilize (**State 1**) with electrocorticogram (ECoG) and electrothalamogram (EThG) recorded continuously until necropsy. Then, isoflurane in N_2_O and O_2_ was discontinued and ventilation with 100% O_2_ was performed for 5 minutes. Another phase (**State 2a**) ensued in which intravenous bolus injection of fentanyl (0,015 mg/kg b.w.) was carried out followed by continuous iv infusion of fentanyl (0.015 mg/kg b.w./h) for 90 minutes (**State 2b**). Next, individual doses of propofol required for the maintenance of deep anesthesia were determined under continuous control of mean ABP (MABP). Propofol was infused intravenously (0.9 mg/kg BW/min for ~7 min) until a burst suppression pattern (BSP) appeared in the ECoG. The depth of anesthesia was subsequently maintained for 25 minutes via propofol administration (~ 0.35 mg/kg b.w./min) (**State 3**). Next, 30% of the propofol dose required for BSP induction was continuously administered over the course of 90 minutes to produce moderate anesthesia. About ten minutes after the onset of the moderate anesthesia period, the first measurement was performed **(State 4)**. **State 5** represented the measurement 60 min after induction of the moderate propofol sedation.

We induced gradual cerebral ischemia as follows^4^. First, the cisterna magna was punctured by a lumbar puncture needle that was fixed in place by dental acrylic resin for elective artificial cerebrospinal fluid infusion/withdrawal to control ICP. Then, the mean ABP was adjusted to about 90 mmHg by the appropriate curbing of the pulmonary trunk diameter with the plastic-coated cerclage. The cerebral perfusion pressure (CPP) was then decreased at 25 mmHg, which was calculated as the difference between MABP and the intracranial pressure (ICP) by appropriate elevation of the ICP. The increase in the ICP was achieved by the infusion of artificial cerebrospinal fluid (warmed to 37 °C) into the subarachnoid space via the punctured cisterna magna. The Cushing response during severe brain ischemia was prevented by the appropriate curbing of the pulmonary trunk diameter with the plastic-coated cerclage to control cardiac output. Finally, the cerclage was opened completely, and the artificial cerebrospinal fluid was withdrawn to reach an ICP < 10 mmHg.

The states of gradual ischemia were maintained for 15 min twice (**States 6 and 8**), interceded by a recovery state (**State 7**) lasting 30 minutes in all animals (P746 - P794) except P739 where it lasted 15 minutes. This was followed by 60 min of recovery (**States 9–12**).

At the end of the experiment, the animals’ brains were perfusion-fixed^2^. Afterward, the head was removed, immersion-fixed, the brain was removed and electrode positions were visually and histologically confirmed.

#### Data acquisition and analyses

Unipolar ECoG and EThG were amplified (DC-EEG-AMPLIFIER, Schwind Medizintechnik, Erlangen, Germany), filtered (time constant was 0.1 second, cut off frequency was 1000 Hz), fed into a multi-channel recording device (GJB Datentechnik Bolten & Jannek GbR, Ilmenau, Germany), stored after A/D conversion continuously on hard disc at 125 Hz and parallel-connected at 2000 Hz for about 300 seconds at predefined states (see below).

#### Cardiovascular and metabolic parameters

ECG, heart rate, MABP, body temperature, arterial and brain venous pH, pCO_2_, pO_2_, O_2_ saturation, glucose, lactate, and hemoglobin values were measured at each State as published^1,2^.

#### Cerebrovascular and cerebral metabolic parameters

At states 1,2,5,6 and 12, whole and regional brain blood flows were measured with colored microspheres^5^ together with brain oxygen extraction (arterial – sagittal sinus blood oxygen content difference, AVDO_2_) and cerebral metabolic rate of oxygen (the product from cortical blood flows and AVDO_2_)^4^.

### Signal analysis methodology

EEGLAB v2019.1 for Linux was used in Matlab R2013b (MathWorks, Natick, MA) to analyze ECoG/EThG data^3^. ECG-derived heart rate variability (HRV) was assessed via a series of automated algorithms that process a waveform recording into a comprehensive multivariate characterization of its degree of variability and complexity metrics, using the Continuous Individualized Multiorgan Variability Analysis (CIMVA) software tool^12^.

Briefly, individual heartbeats were identified from the ECG waveform, deploying commonly used QRS delineation algorithms, and a time series of R-peak to R-peak time intervals (RRI) was formed. Movement artifacts, noise, disconnections, and saturations were automatically identified, and a beat-by-beat signal quality index was derived, using continuity and morphology analyses. The RRI time series was then filtered to exclude abnormal beats and the signal complexity and degree of variability were assessed using the cleaned RRI time series. HRV metrics were tracked over time using a moving window analysis (5-minute windows with a 50% overlap between windows). A comprehensive set of linear and nonlinear variability metrics were calculated within each window, as each technique provides a unique perspective on the data and no single method can provide a complete characterization of the biologic signals^13,14^. Variability metrics included measures characterizing the statistical properties (e.g., standard deviation, RMSSD), the informational complexity (e.g., entropy measures), the pattern of variations across timescales (e.g., fractal measures, power-law exponents) or the energy contained in the signal (e.g., spectral measures). The output of the variability analysis was a multivariate representation of variability tracked over time^15,16^. The variability metrics were then averaged over the different periods of interest.

## Data Records

The data structure and annotation are as follows. 16 channels are given at a sampling rate of 2000 Hz and a recording duration of about 300 s per state each containing the following channels.

Channel 1 Temp re, the rectal temperature in Celsius

Channel 2 ABP, arterial blood pressure measured from the abdominal aorta

Channel 3 ECG, electrocardiogram

Channel 4 CVP, central venous pressure

Channel 5 ICP, intracranial pressure

Channels 6–14 ECoG, frontal, parietal, central, temporal, and occipital regions

Channels 15, 16 EThG from RTN and LD nucleus

File duration per state is 300 s. Sampling rate: 2000 Hz. Note that while Fig. 1 and Table 1 show the entire instrumentation approach, ECoG channel 3 was of poor quality in most instances due to intermittent technical problems (random faults) and an amplifier breakdown. We hence removed this channel from the dataset presented. That means that the left parietal ECoG (channel 3) is not included in the dataset.

**Table 1:** Stereotactic coordinates*.

Position	Lateral of sagittal suture	Anterior (a) / Posterior (p) of bregma	Depth from the dura	Equivalent according to 10/20 single plane projection of the head^37,38^
Frontal ECoG, Ch. 6 & 7 (left & right)	12 mm	a 30 mm		Fp1, Fp2
Parietal ECoG, Ch 8 (left^#^ & right)	12 mm	a 20 mm		F3^#^, F4
Central ECoG, Ch. 9 & 10 (left & right)	12 mm	a 10 mm		C3, C4
Temporal ECoG, Ch. 11 & 12 (left & right)	24 mm	p 10 mm		T5 = P7, T6 = P8
Occipital ECoG, Ch. 13 & 14 (left & right)	12 mm	p 10 mm		P3, P4
EThG, Ch. 15 (RTN = Nucl. reticularis thalami)	9 mm	a 2 mm	24 mm	
EThG, Ch. 16 (LD = Nucl. dorsolateralis thalami)	5 mm	p 2 mm	20 mm	

* Reference: Nz

^#^, excluded from the dataset due to poor signal quality.

**Fig. 3:**
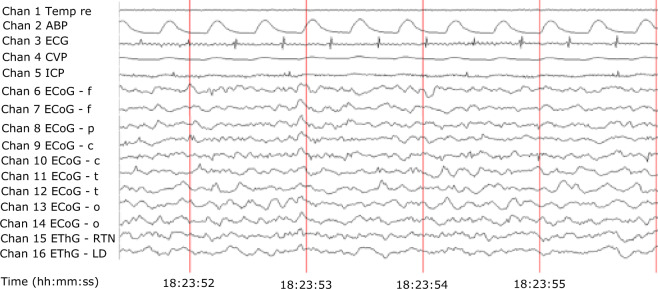
Representative raw recording during data acquisition in Watisa^®^ software (State 1). Sixteen channels are given at a sampling rate of 2000 Hz (Channel (Chan) 1 - Temp re, rectal temperature [°C]; Chan 2 - ABP, arterial blood pressure [mm Hg]; Chan 3 - ECG, electrocardiogram [mV]; Chan 4 - CVP, central venous pressure [mm Hg]; Chan 5 - ICP, intracranial pressure [mm Hg]; Chan 6–14 - ECoG, frontal (f), parietal (p), central (c), temporal (t), and occipital (o) regions [µV] (odd channel number, left-hand side; even channel number, right-hand side); Chan 15, 16 - EThG, RTN, LD nucleus [µV]).

The sample raw recording is shown in Fig. 3.

All animals underwent gradual ischemia. The two groups are defined with respect to their exposure to propofol sedation. The experimental group comprises N = 6 animals (P728, 737, 738, 743, 746, 752). They experienced propofol burst suppression followed by moderate propofol sedation prior to gradual ischemia. The propofol sham group comprises N = 5 animals (P739, 749, 753, 791, 794). They did not receive propofol. Instead, they were continued on fentanyl analgosedation prior to gradual ischemia.

The animal P728 experienced an experimental mishap during the first gradual ischemia stage (state 6). The recordings are presented up until state 4 and can be grouped with other animals’ data for the respective states 1 through 4. The animal P738 demised prematurely during the second gradual ischemia (state 8). Consequently, the data is presented up until state 6 and can also be grouped and studied together up until this point. Here, special consideration should be made for the potential incipient deterioration leading up to the early demise which represents a point of interest.

In addition to the raw data, we deposited the corresponding, time-matched arterial and cerebral-venous measurements of blood gases, electrolytes, metabolites, cardiovascular, and cerebrovascular as well as CMRO_2_ data. The exact list of the types of data is outlined in Table 2.

**Table 2:** Parameters collected during each state of the experiment.

Arterial blood data	body temperature [°C]^a^ hemoglobin [mmol/l] oxygen saturation, Carboxy-hemoglobin (%), Methemoglobin, Oxygen content, Oct (Vol%) (%)^b^ [%] pH pCO_2_ [mmHg] pO_2_ [mmHg]^c^ acid base excess [mmol/l]^c^ K + , Na + , glucose, lactate^d^
Sagittal sinus venous blood data	hemoglobin [mmol/l] oxygen saturation, Carboxy-hemoglobin (%), Methemoglobin, Oxygen content, Oct (Vol%) (%)^b^ [%] pH, pCO_2_ [mmHg], pO_2_ [mmHg]^c^ acid base excess [mmol/l]^c^ K + Na + , glucose, lactate^d^
Cardiovascular data	arterial blood pressure, ABP (mm Hg)^e^cardiac output (ml/min * kg body weight)^f^
Cerebrovascular data	CBF_Whole brain, wCBF (ml/100 g*min)^f^
	cerebral perfusion pressure (mm Hg) = ABP – intracranial pressure, ICP^g^
	cerebral metabolic rate of oxygen, CMRO_2_ (ml/100 g*min) = wCBF × arterial-brain venous difference of Oct, (AVDO_2_)
Brain regional blood flow (ml/100 g*min)	Thalamus^f^
	Frontal cortex^f^
	Parietal cortex^f^
	Temporal cortex^f^
	Occipital cortex^f^

^a^Rectal temperature probe (Digi-Sense, Scanning thermocouple thermometer, Cole-Parmer GmbH Wertheim, Germany).

^b^Hemoxymeter OSM2, Radiometer, Copenhagen, Denmark.

^c^ABL50 Blood Gas Analyzer, Radiometer, Copenhagen, Denmark.

^d^Electrolyte and metabolite analyser, model EML105, Radiometer, Copenhagen, Denmark.

^e^DC-Bridge amplifiers, Hugo Sachs Elektronik, March-Hugstetten, Germany.

^f^Colored microsphere technique (according to^5^).

^g^Camino V420, Camino Laboratories, San Diego, USA.

All data have been deposited on Figshare in BIDS format, as European Data Format (EDF) files with uniquely identifiable animal ID and states numbers as well as in the structure of EEGLAB’s STUDY object which contains the specific annotation of all file names and experimental states as well as the more general states (sedation, ischemia, recovery) for any future analyses. The data can be located under the 10.6084/m9.figshare.7834442.v9 and 10.18112/openneuro.ds003380.v1.0.0^17,18^.

## Technical Validation

### Brain electrical activity

We present the approach and findings of signatures of global and brain-regional changes in ECoG-derived independent component activity during sedation, ischemia, and recovery states. Table 2, bold font, lists the animal IDs and experimental states we selected for technical validation. We considered states 1–5 combined as sedation, states 6 and 8 as ischemia, and states 9–12 as post-ischemic recovery. Using the STUDY design feature of EEGLAB in Matlab, we conducted statistical analyses on a subgroup of six animals (P737, P739, P752, P753, P791, P794) as follows:

1. read raw data (2,000 Hz sampling rate);
2. select 10 ECoG channels;
3. remove DC offset;
4. resample at 100 Hz;
5. a bandpass filter with FIR 1–40 Hz and save as *.set files (also shared on figshare)^17^
6. map ECoG channel locations for better visualization using Nz as reference (.loc file shared on FigShare;^17^ cf. Table 1)
7. create STUDY in EEGLAB; this approach permits statistical level inferences across animals for state-specific changes common to all subjects
8. compute independent components for all animals and states in the STUDY
9. identify IC clusters (using k-means approach) that differentiate sedation, ischemia, and recovery states.

We deployed EEGLAB software suite (http://sccn.ucsd.edu/eeglab)^3,19,20^ available as Matlab/Octave add-on, or a pre-compiled open-source package for Windows, Mac, and Linux operating systems to conduct technical validation of our data set and to demonstrate some initial findings of interest for future studies. The technical advantage of this approach is that this software package is open source and readily available online for the major operating systems.

We focused on the representative experimental states 2 (baseline), 6 (acute gradual ischemia), and 12 (60 min recovery) for all subjects using the STUDY functionality of EEGLAB which allows studying all subjects at once compared the event-related potential (ERP) responses within the group. ERP represented the response to the states of the experiment (Table 3). To facilitate the computation, we downsampled the data to 100 Hz and focused on the ECoG channels for EEGLAB-based analysis which allowed us to map the channels according to 10/20 using a channel location file (see Figshare^17^). Future studies on this data set could consider the full bandwidth ECoG/EThG information contained in the recordings and include EThG channels in the investigations.

**Table 3:** Review of experimental stages and the respective available data sets*.

Experimental stage number	Experimental stage	
**state1**	Isoflurane	P_728, **P_737**, P_738, **P_739**, P_743, P_746, P_749, **P_752, P_753**, **P_791, P_794**
***state2***	Fentanyl	P_728**, P_737**, P_738**, P_739**, P_743, P_746, P_749, **P_752, P_753**, **P_791, P_794**
state2.5	90 min post-fentanyl	P_739, P_753
**state3**	Propofol	P_728**, P_737**, P_738, P_743, P_746, **P_752**
**state4**	Moderate sedation - immediate measurement	P_728**, P_737**, P_738, P_743, P_746, **P_752**
**state5**	60 min post-moderate sedation	**P_737**, P_743, P_746, **P_752**
state5.5	Fentanyl directly pre-ischemia	P_794
**state6**	1^st^ ischemic phase: gradual ischemia	P_738*, **P_739**, P_746, P_749*, **P_752, P_753**
state 7a^#^	15 min recovery post-ischemia (first period)	P_739, P_746, P_749, P_752, P_753, P_791, P_794
state 7b^#^	30 min recovery post-ischemia (first period)	P_746, P_749, P_752, P_753, P_791, P_794
**state8**	2^nd^ ischemic phase: gradual ischemia	**P_739**, P_746, P_752*, P_753, P_791, P_794*
**state9**	15 min recovery post-ischemia	**P_737**, P_743 (after single ischemia period); **P_739**, P_746, P_749, **P_752, P_753**, **P_791, P_794** (after second ischemia period)
**state10**	30 min recovery post-ischemia	P_746, P_749, **P_752, P_753**, **P_791, P_794**
**state11**	45 min recovery post-ischemia	**P_737**, **P_739**, P_743, P_749, **P_752, P_753**, **P_791, P_794**
**state12**	60 min recovery post-ischemia	**P_737**, P_743, P_749, **P_752, P_753**, **P_791, P_794**

Bold font: animals and states selected for the analyses presented in the Technical validation section.

^#^the recordings of the state 7 are available at 100 Hz sampling rate, rather than 2000 Hz as all other states; all recordings are provided in 5 min duration.

*for these animals, at 1^st^ and 2^nd^ stages of ischemia we provided 5 min files - in keeping with the rest of the dataset - representing the final stage of each ischemia episode. Details including any artifacts observed, for each file and for each channel, are provided in the auxiliary spreadsheet, also available on FigShare^17^.

We performed the independent component analysis (ICA) on animals identified in Table 3 followed by wavelet time-frequency and cross-coherence analyses on the identified independent components.

**Fig. 4:**
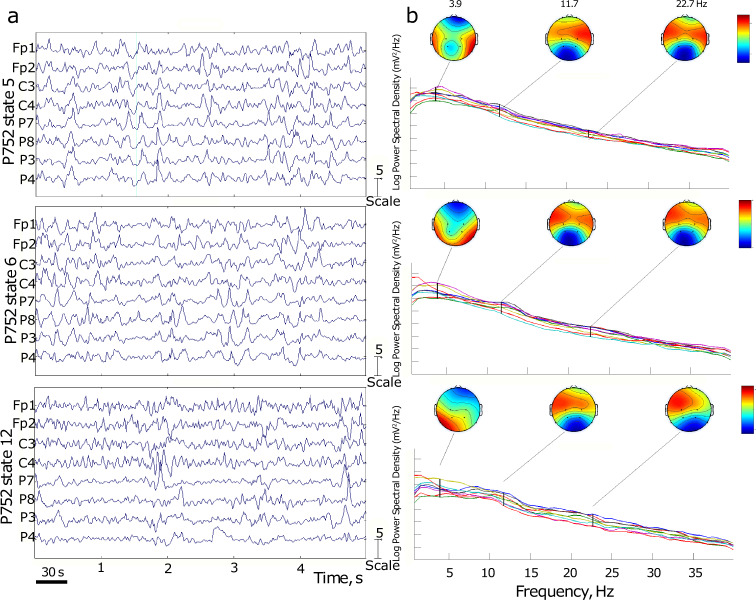
Representative findings for sedation (state 5, moderate propofol sedation), ischemia (state 6, ischemia) and recovery (state 12, 60 min recovery)*. For each state, we show the raw ECoG tracings (Fig. 4a, normalized and DC removed), corresponding topological power spectrum maps (Fig. 4b) showing gradual suppression of activity with peaks at 4, 12, and 23 Hz. *Note that all axes are set identically for easy comparison except the topological and time-frequency transform heat maps. These are individually optimized in range for best viewing experience.

**Fig. 5:**
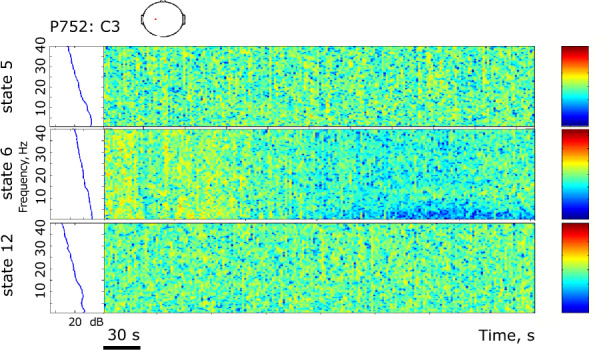
Representative findings for sedation (state 5, moderate propofol sedation), ischemia (state 6, ischemia) and recovery (state 12, 60 min recovery)*. For each state, we show the time-frequency representation using wavelet transform on the channel 5 (i.e., C3). *Note that all axes are set identically for easy comparison except the topological and time-frequency transform heat maps. These are individually optimized in range for best viewing experience.

**Fig. 6:**
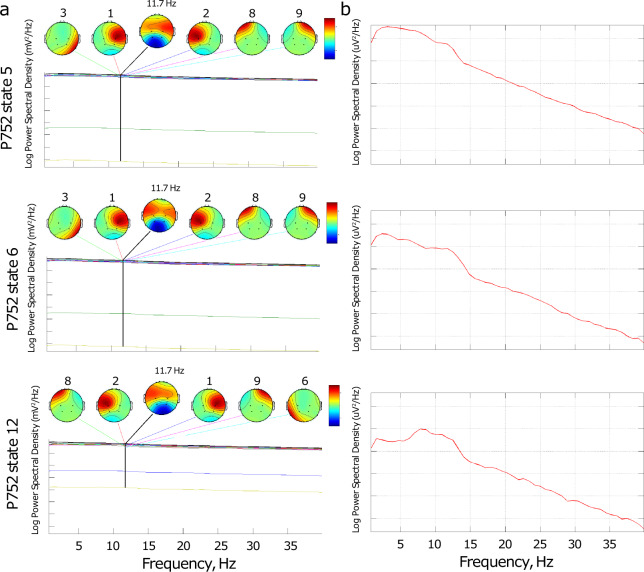
Representative findings for sedation (state 5, moderate propofol sedation), ischemia (state 6, ischemia) and recovery (state 12, 60 min recovery)*. For each state, we show in Fig. 6a the independent component (IC) analyses with focus on 12 Hz activity (one of the clearest consistent peaks in the entire group) for the five top ICs in each state and the representative IC2 power spectrum (Fig. 6b), again showing the overall reduction of activity. *Note that all axes are set identically for easy comparison except the topological and time-frequency transform heat maps. These are individually optimized in range for best viewing experience.

**Fig. 7:**
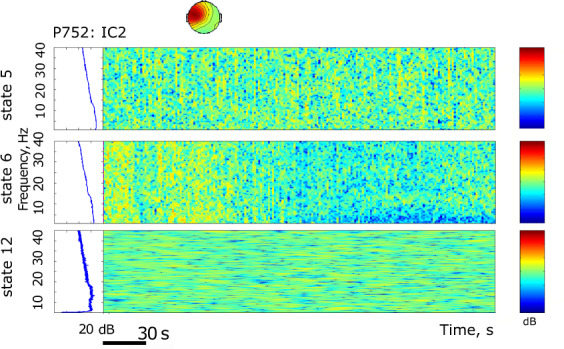
Representative findings for sedation (state 5, moderate propofol sedation), ischemia (state 6, ischemia) and recovery (state 12, 60 min recovery)*. For each state, we show the time-frequency representation using wavelet transform on the IC2 corresponding to the power spectrum representation in Fig. 6b. *Note that all axes are set identically for easy comparison except the topological and time-frequency transform heat maps. These are individually optimized in range for best viewing experience.

**Fig. 8:**
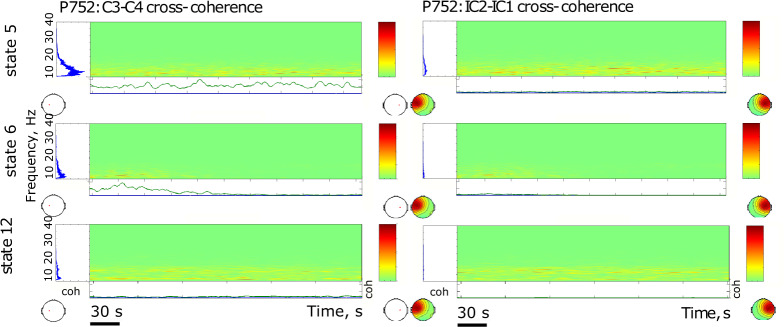
Representative findings for sedation (state 5, moderate propofol sedation), ischemia (state 6, ischemia) and recovery (state 12, 60 min recovery)*. For each state, we show the cross-coherence representations of bihemispheric coupling between C3-C4 and IC2 - IC1. *Note that all axes are set identically for easy comparison except the topological and time-frequency transform heat maps. These are individually optimized in range for best viewing experience.

**Fig. 9:**
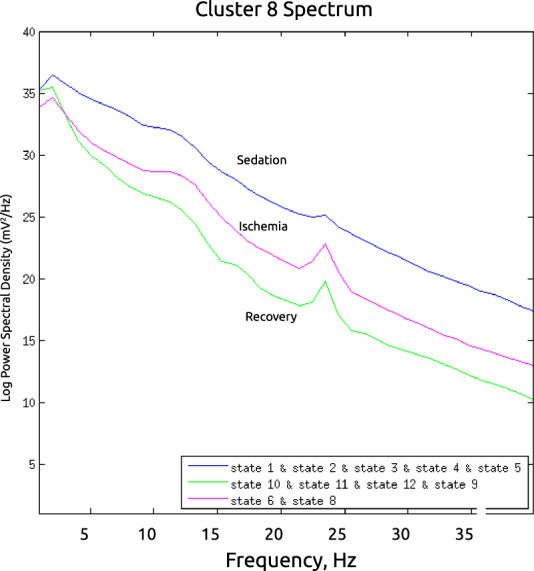
Group-level separation of three states, sedation, ischemia and recovery, in the set of six animals (P737, P739, P752, P753, P791, P794) based on nine clustered independent components computed from eight ECoG channels. We observe the gradual reduction of spectral power between the experimental states representing sedation (states 1–5, blue line), ischemia (states 6 and 8, red line), and recovery (states 9b − 12, green line). We observe the peaks around 12 and 23 Hz as seen in the representative examples in Figs. 4–8.

The results are presented in Figs. 4–9. The analysis of the raw data (Fig. 4a) revealed the activity to be concentrated in the delta - alpha band range which experienced most of the reduction in spectral power in the individual channels (Figs. 4b, 5–7) as well as in cross-coherence (Fig. 8) due to ischemia and during recovery. Following ICA, we observed an overall reduced cross-coherence in IC2 - IC1 compared to raw channel C3 - C4 analysis, but still following a similar pattern throughout states 5, 6, and 12 (Fig. 8). As expected, a portion of the observed coherence is accounted for by volume conduction effects which are removed by ICA. As evidenced by the group analysis results of which are shown in Fig. 9, 60 min recovery did not suffice to restore the global reductions spectral power activity seen during the ischemic states.

Ischemia reduces total power across the spectrum going from sedation states over to ischemia and then recovery, however, during recovery the bihemispheric coupling is re-established. A combination of spectral power characteristics and coupling dynamics can be used to distinguish ischemia-induced loss in spectral power and post-ischemic recovery. Clustered ICA also distinguishes these states.

In the example of P752, we focus on the representative states 5 (60 min into moderate propofol *sedation*), 6 (gradual *ischemia*), and 12 (60 min post-ischemic *recovery*). For each state, we show the following:

1. the raw ECoG tracings (Fig. 4a, normalized and DC removed), corresponding topological power spectrum maps (Fig. 4b) showing gradual suppression of activity with peaks at 4, 12, and 23 Hz, and the time-frequency representation using wavelet transform on the channel 5 (i.e., C3; Fig. 5);
2. the independent component (IC) analyses (Fig. 6a) with focus on 12 Hz activity (one of the clearest consistent peaks in the entire group) for the five top ICs in each state; representative IC2 power spectrum (Fig. 6b), again showing the overall reduction of activity and the corresponding time-frequency representation using wavelet transform on the IC2 (Fig. 7);
3. cross-coherence representations of bihemispheric coupling between C3-C4 and IC2 - IC1 (Fig. 8)
4. Group analysis (Fig. 9) showing the gradual reduction of spectral power between the experimental states representing sedation (states 1–5, blue line), ischemia (states 6 and 8, red line), and recovery (states 9–12, green line). We observe peaks around 12 and 23 Hz which triggered our focus on these frequencies in the representative examples shown in Figs. 4–8.

Overall, this finding shows that ischemia and recovery states can be distinguished globally using such an ICA approach. As a proof-of-concept, this analytical approach in EEGLAB demonstrates the potential of the presented experimental data set to yield new pathophysiological insights into global and brain-regional responses to sedation, gradual ischemia, and post-ischemic recovery.

### Cardiovascular activity

To demonstrate an approach to use the present dataset for studying the unique features of cardiac electrical activities during sedation, ischemia, and recovery stages, we exported the ECG channel (Ch. 3) at 1000 Hz as EDF files for the same animals for which we presented the above approach on the ECoG data (see Figshare for ECG-ECoG data and extended results)^17^.

**Fig. 10:**
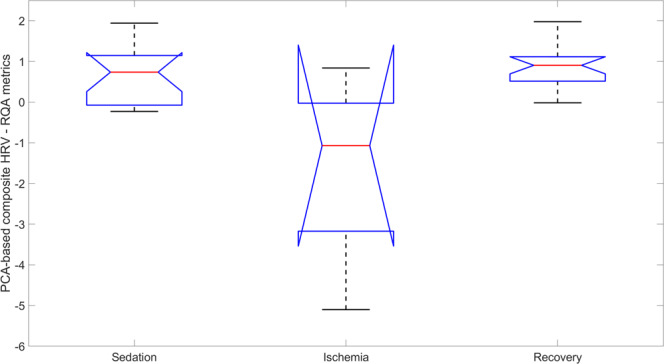
Group level overview of the behavior of the ECG-derived multi-dimensional heart rate variability (HRV) measures. Principal component analysis (PCA) was used to separate the states of sedation, gradual ischemia and recovery and shows the best performing PCA over RQA (recurrence quantification analysis) measures of HRV. Note the general drop of HRV during ischemia and recovery during the period of post-ischemic recovery. The notched boxplots representation is helpful in visualizing the difference in the median; two medians are significantly different at the 5% level if their intervals (extremes of the notches) do not overlap^39^.

Briefly, first, we computed HRV measures from the ECG channel using the approach described in Section 3^15,16^. Next, we grouped the findings according to the three main states of the experiments into sedation, ischemia and recovery following the same approach as in Fig. 9. Finally, we visualized the changes in the HRV measures in Fig. 10 using the principal component analysis (PCA). The complete findings on all HRV measures are shown in the auxiliary data accessible on FigShare^17^. The PCA demonstrates how the array of HRV measures dynamics can separate the states of sedation, ischemia, and recovery.

In detail, the HRV measures used are summarized in Online-only Table 1. First, we normalized HRV measures by the mean heart rate (HR) when calculating the variability to minimize the effect of the different HR levels in the different states. Second, we standardized each metric and grouped the variability metrics in broad categories according to what they represent (Fractal, Chaotic, Entropies, Spectral, Time Domain, Poincare, Symbolic Dynamics, RQA) and harmonized the meaning across metrics so that small values represent less variability or less complexity and large values the opposite. We did so by inverting the variability metrics or the absolute value of the variability metrics, when appropriate. For each category of metrics, we then performed a PCA transformation and reconstructed a composite HRV metric for each category, based on the N components explaining 90% of the variance in the data. Finally, we created notched boxplots per group. Such boxplots give an estimate of the confidence interval around the median, whereby two medians are significantly different at the 5% level if their intervals (extremes of the notches) do not overlap. We also prepared the PCA boxplot based on ungrouped metrics, i.e., all 54 metrics in one plot (see FigShare^17^).

Overall, very few differences in PCA-based HRV measures were significant, which is expected given the small sample size. RQA-based HRV measures did stand out (Fig. 10) and this is consistent with our findings implicating these HRV measures in reflecting the intrinsic cardiac function which was affected severely by the induced ischemia^21,22^.

### Limitations

To perform technical validation, we selected six animals for sedation, five for ischemia and six for recovery periods. However, within these broad experimental periods, not all individual animals were represented equally for each experimental state. This may cause a data imbalance issue affecting the statistical results, in particular given the small sample size. With our EEGLAB STUDY design, we pursued the intention of quantifying general trends characterizing differences between the periods of sedation, ischemia and recovery. We suggest that this is adequately demonstrated through presented ECoG and HRV analyses and leave a more in-depth analysis to future studies on this dataset.

## Usage Notes

For thalamocortical and cortico-cortical communication, sedation-dependent linear and nonlinear coupling dynamics have been reported^1^, but not yet characterized under conditions of gradual ischemia as a potential biomarker of brain state and recovery. Studies in rats have shown that global brain hypoxia-ischemia affects long-term information processing in thalamic circuitry and the transfer of sensory information in thalamocortical networks^23,24^. Newborn mice exposed to ischemic insult also suffer from the increased vulnerability of thalamocortical circuitry^25^. There is less data on thalamocortical responses to ischemia from larger mammals with stronger resemblance of brain maturity, developmental profile, and injury patterns, such as sheep or pig^26^. The present dataset may help to close this data gap and yield new insights into monitoring, early detection, recovery of ischemic and post-ischemic brain states, in particular, thalamocortical communication which are important to help restore long-term brain health.

Anesthesia-induced changes in brain electrical activity, in particular, due to the dedicated GABA A receptor-mediated effects of propofol on the RTN, have been used to model and study changes in consciousness and behavioral state activity^27–30^. In this experimental design, we benefit from propofol sedation with electrode placement in the RTN, which, as discussed, is particularly amenable to linking drug-induced agonism on GABA A receptors of RTN and the sedation depth^1^. The combination of propofol sedation with subsequent ischemia data from ECoG and EThG, including Nucl. reticularis thalami, yields a rich dataset to study patterns of thalamocortical communication under conditions of sedation, ischemia, and recovery.

The present dataset has been acquired at a 2,000 Hz sampling rate and is hence amenable to studies of the properties of high-frequency oscillations under conditions of various sedation regimes, gradual ischemia, and recovery periods^31–33^. Of great interest, thanks to EThG recordings, it may be possible to relate the ECoG patterns of spontaneous high-frequency oscillations to their thalamic contributions in EThG.

In this data-oriented manuscript, we used EEGLAB for the demonstration of the technical rigor, data quality, and reproducibility. While rich in functionality and open source, in future studies of this dataset the EEGLAB application could be well-complemented by additional analytical approaches, also available open-source, such as the JIDT software package^34^. (https://github.com/jlizier/jidt/) JIDT is Java-based, offers GUI, requires no installation, and runs on all major platforms. It also can be integrated with Matlab, Python, or R, among others. Features, of interest to this dataset that are available with JIDT, include mutual information and transfer entropy. It would be of interest to compute these before and after ICA as well as after subtracting IC expecting the bi-channel / bivariate information measures to decrease after ICA and re-increase after removing ICs. Using JIDT software, one can also determine cross-entropy measures for ECoG - EThG channels prior to and after IC computation.

Finally, the simultaneous availability of the cardiovascular and brain electrical data lends itself to studying joint dynamics of, for example, ECG and ECoG or EThG. The relationships between these time series have been reported^35,36^, sporadically, but much remains to be done to establish the physiological framework for these relations and to explore the biomarker potential of such multivariate EEG - ECG analyses. We leave this exciting direction of research to future studies using this data set.

## Data Availability

EEGLAB has been used which is available as open-source. CIMVA documentation is available online: https://ohridal.org/cimva/CIMVA-Core-Description.pdf. No proprietary code has been deployed in this study.

## Online-only Table

**Online-only Table 1:** Heart rate variability approach.

Fractal			
fFdP	Fano factor distance from a Poisson distribution	Describes fractal-like point processes via a modified estimate of the Fano factor, based on the average distance to a Homogeneous Poisson Point Process.	small = less complex
aFdP	Allan factor distance from a Poisson distribution	Describes fractal-like point processes via a modified estimate of the Allan factor based on the average distance to a Homogeneous Poisson Point Process.	small = less complex
MultiFractal_c1	MultiFractal spectrum cumulant of the first order	Maximum of the multifractal spectrum that can be viewed as the most common variability within a window, similar to a global variability.	small = more variability
MultiFractal_c2	MultiFractal spectrum cumulant of the second order	Width of the multifractal spectrum i.e. how variability departs from C1 value. Small C2 = variability changes little over time. High C2 = variability of the signal can differ greatly over time	small = more variability
Correlation dimension	Correlation dimension	Overall complexity; related to fractal dimension; minimum number of variables required to characterize system dynamics.	small = less complex
DFA Alpha 1	Detrended Fluctuation Analysis: α exponent	Scaling analysis method to represent correlation properties. α≈1: fractal-like temporal structure; α = 0.5 uncorrelated data (white noise); α = 1.5 Brownian noise (smooth fluctuations).	closer to 0.5 = roughest; closer to 1.5 = smoothest; healthy ≈ 1
DFA Alpha 2	Detrended Fluctuation Analysis: α1 exponent	Scaling analysis method to represent short-term correlation properties. α≈1: fractal-like temporal structure; α = 0.5 uncorrelated data (white noise); α = 1.5 Brownian noise (smooth fluctuations). Thought to reflect baroreceptor reflex or parasympathetic modulation.	closer to 0.5 = roughest; closer to 1.5 = smoothest; healthy ≈ 1
DFA AUC	Detrended Fluctuation Analysis: Area Under the Curve	Area Under the DFA curve estimating the total variance of the signal across time scales	small = low variability
Power Law Slope LombScargle	Power Law Slope (LombScargle method)	Slope of the linear portion of the power spectrum on a log-log plot. Represents long term scaling of fractal like processes with long range dependence (i.e. 1/f power-law exponent)	small = less complex
Hurst exponent	Hurst exponent	Index of long-range dependence and related to the fractal dimension of a system i.e. the minimum number of degrees of freedom of the dynamical system.	small = higher fractal dimension
eScaleE	Embedding Scaling exponent	Estimates fractal scaling by looking at how much the covariance of embedded vectors changes with embedding dimension. It relates to the Hurst exponent.	small = less complex
AsymI	Multiscale time irreversibility asymmetry index	Degree of temporal asymmetry and lack of invariance of the statistical properties of a signal over multiple scales; Pathologic signals are more symmetric than healthy ones.	small = less complex
Chaotic			
Largest Lyapunov exponent	Largest Lyapunov exponent	Represents the average exponential growth rate of the distance between two neighbouring points in the dynamical system trajectory as time evolves. Measure of predictability, entropy rate, chaotic behavior. It is positive for chaotic data.	small = less complex/chaotic
SDLEalpha	Scale dependent Lyapunov exponent slope	Multiscale complexity measure estimating the main slope of the log-log plot of Lyapunov exponents at multiple scales, which characterizes the speed of loss of information i.e. the uncertainty involved in predicting the value of a random variable. Initial slope (lower scales) typically indicates noisy dynamics.	small (steeper descending slope) = more complex
SDLEalpha2	Scale dependent Lyapunov exponent slope (after break)	Multiscale complexity measure estimating the main slope of the log-log plot of Lyapunov exponents at multiple scales, which characterizes the speed of loss of information i.e. the uncertainty involved in predicting the value of a random variable. Typically the second slope is flat and indicates chaotic regime.	if flat = chaotic regime
SDLEmax	Scale dependent Lyapunov exponent max value	Multiscale complexity measure estimating the maximum of Lyapunov exponents across multiple scales. The maximum typically occurs at small scales and is related to entropy measures.	small = less complex
gcount	Grid transformation feature: grid count	Trajectory of a dynamical system is represented on a discrete grid and the number of “visited” pixels is counted. Represents the degree of complexity or chaotic dimension.	small = less complex
sgridAND	Grid transformation feature: AND similarity index	Estimates self-similarity (regularity of a signal) between discretized delayed versions of dynamical system trajectories. Measures both the spread of trajectories as well as their similarity between two time instants.	small = less similar
sgridTAU	Grid transformation feature: Time delay similarity index	Estimates self-similarity (regularity of a signal) between discretized delayed versions of dynamical system trajectories.	small = less similar
**Entropies**			
shannEn	Shannon Entropy	Average information content of a signal	small = less complex
Sample entropy	Sample Entropy	Represents the signal unpredictability or regularity within short time segments by estimating the entropy rate	small = less complex
QSE	Quadratic Sample Entropy	Normalized version of the sample entropy (with respect to the tolerance window); Represents the signal unpredictability or regularity within short time segments by estimating the entropy rate	small = less complex
Multiscale Entropy	Multiscale Entropy	Represents signal unpredictability/regularity within short time segments (akin to sample entropy) but over multiple scales	small = less complex
KLPE	Kullback Leibler permutation entropy	Deviation from randomness. With increasing complexity of the time series, KLPE decreases until it reaches zero for noise. Higher values indicate better predictability of the system.	small = more complex
Control entropy	Control Entropy	Entropy measure designed to be robust to non-stationary signals; Sample entropy of the successive differences	small = less complex
ARerr	Predictive feature: error from an autoregressive model	Measure of predictability	small = low variability
**Spectral**			
LF Power LombScargle	LF Power (Lomb-Scargle method)	Power contained in the low frequency band (0.04–0.2 Hz for fetal recordings) of the ECG spectrum. Reflects both sympathetic and vagal activity, and blood pressure regulation via baroreceptors.	small = less power
HF Power LombScargle	HF Power (Lomb-Scargle method)	Power contained in the high frequency band (0.2–2 Hz for fetal recordings) of the ECG spectrum. Mostly reflects vagal modulation of HR and is related to the respiratory cycle.	small = less power
LF/HF ratio LombScargle	LF/HF ratio (Lomb-Scargle method)	Represents sympathetic and parasympathetic modulation but its interpretation is experiment dependent and unclear in general.	small = less power
VLF Power LombScargle	VLF Power (Lomb-Scargle method)	Power in the Very Low Frequency Band. Thought to relate to thermoregulation and to be sympathetically mediated	small = less power
**RQA**			
pD	RQA: percentage of determinism	% of recurrent points forming diagonal lines, with a minimum of two adjacent points (deterministic). Measures predictability	small = more chaotic
pDpR	RQA: determinism/recurrences	ratio of %Determinism over %Recurrence. Quantifies transition/nonstationary periods in a system.	small = more stationary
pL	RQA: percentage of laminarity	Laminarity detects laminar states (chaos-chaos transitions) and rapid changes in RRI	small = more complex
pR	RQA: percentage of recurrences	Global measure of recurrence (% of plot filled with recurrent points). Periodic dynamics have higher % of recurrence than aperiodic dynamics.	small = more complex
dlmax	RQA: maximum diagonal line	Maximal length of the diagonal structures in a recurrence plot, representing exponential divergence of the trajectories. Detects transitions from periodic to chaotic behavior and Inversely related to the largest lyapunov exponent.	small = more chaotic
dlmean	RQA: average diagonal line	Average length of the diagonal structures in a Recurrence Plot. Mean prediction time of the system.	small = more chaotic
sedl	RQA: Shannon entropy of the diagonals	Rough measure of the information content of the trajectories (diagonal lines) on a Recurrence plot.	small = less complex
vlmax	RQA: maximum vertical line	Max length of vertical lines on a Recurrence plot. Information about the time duration of the laminar states and marker of intermittency	small = more chaotic
**Time domain**			
Mean	Mean of RR-intervals	Sample mean of the RR interval time series.	small = low variability
Standard Deviation	Standard Deviation	Standard deviation of Normal-Normal (NN) intervals.	small = low variability
RMSSD	Root mean square of successive RR interval differences	Represents beat-to-beat variance in HR and estimates vagally mediated changes in HRV. Mathematically equivalent to Poincare SD1	small = low variability
Coefficient of variation	Coefficient of variation	Standard deviation normalized by the mean	small = low variability
histSI	Similarity index of the distributions	Similarity index between the statistical distribution of two consecutive data blocks (0: lowest similarity or highest variability; 100: highest similarity or lowest variability)	small = high variability
Complexity	Hjorth’s Form Factor (Complexity)	Measure of “excessive details with reference to sine wave”, assesses complexity in the signal; Complexity of the first order. Sensitive to noise. Can be used to detect arrhythmias?	small = less complex
**Poincaré**			
Poincaré SD1	Poincaré plot SD1	Poincaré plot standard deviation perpendicular the line of identity. Represents short interval variations and reflects parasympathetic index of sinus node control. Mathematically equivalent to RMSSD.	small = low variability
Poincaré SD2	Poincaré plot SD2	Poincaré plot standard deviation along the line of identity. Represents long interval variation and is linked to both parasympathetic and sympathetic tones. Related to Standard Deviation	small = low variability
CSI	Poincare plot Cardiac Sympathetic Index	Ratio between SD2 and SD1, represents unpredictability of the RR time series	small = more random/scattered
CVI	Poincare plot Cardiac Vagal Index	Log of the Area of the ellipse fitted to Poincare plot: Total variability	small = low variability
**Symbolic dynamics**			
SymDce_2	Symbolic dynamics: modified conditional entropy, non-uniform case	Characterizes entropy rate	small = less complex
SymDfw_2	Symbolic dynamics: forbidden words, non-uniform case	High number of forbidden words indicates a more regular behaviour of time series	small = more complex
SymDp0_2	Symbolic dynamics: percentage of 0 variations sequences, non-uniform case	Patterns with no variation i.e. all the symbols are equal. Thought to reflect cardiac autonomic modulation, predominantly sympathetic modulation.	small = more complex
SymDp1_2	Symbolic dynamics: percentage of 1 variations sequences, non-uniform case	Patterns with one variation i.e. two consecutive symbols are equal and the remaining one is different	small = less complex
SymDp2_2	Symbolic dynamics: percentage of 2 variations sequences, non-uniform case	Patterns with two variations (either like or unlike variations). Thought to reflect cardiac autonomic modulation, predominantly parasympathetic modulation.	small = less complex
SymDse_2	Symbolic dynamics: Shannon entropy, non-uniform case	Characterizes entropy, i.e., complexity of the pattern distribution.	small = less complex
